# Reported injury, hospitalization, and injury fatality rates among New Jersey adolescent workers

**DOI:** 10.1186/s40621-019-0216-9

**Published:** 2019-08-19

**Authors:** Daniel Uhiara, Derek G. Shendell, Marija Borjan, Judith M. Graber, Koshy Koshy, Margaret Lumia

**Affiliations:** 1 Rutgers School of Public Health (SPH), NJ Safe Schools Program, 683 Hoes Ln. West Suite 399, Piscataway, NJ 08854 USA; 2 Department of Epidemiology, Rutgers SPH, Piscataway, NJ USA; 3 Department of Environmental and Occupational Health, Rutgers, SPH, Piscataway, NJ USA; 4 Environmental and Occupational Health Sciences Institute, Rutgers Biomedical and Health Sciences, Piscataway, NJ USA; 5 NJ Department of Health, Occupational Health Surveillance, Trenton, NJ USA

**Keywords:** Adolescents, Youth workers, Work-related fatality, Injury-related hospitalizations, Injury surveillance

## Abstract

**Background:**

Workplace injuries are a public health concern, including among adolescents and young adults. Secondary school career-technical-vocational education related injuries are mandated by code under jurisdiction of New Jersey Department of Education to be reported online to New Jersey Safe Schools Program. These are the only U.S. law-based surveillance data for young workers in secondary school career-technical-vocational education. New Jersey Department of Health’s hospitalization and fatality records provide additional information about other secondary school career-technical-vocational education and non- secondary school career-technical-vocational education related injuries not necessarily reported to New Jersey Safe Schools Program. This report compared data available to the New Jersey Department of Health and New Jersey Safe Schools Program on injuries among young workers ages 14–21 years.

**Methods:**

Annual work-related hospitalizations, 2007–2016, were abstracted from hospital discharge data. Denominator data from the U.S. Bureau of Labor Statistics was used to estimate annual crude rate of hospitalizations per 100,000 employed persons. Hospitalization rates were stratified by demographic data from the U.S. Bureau of Labor Statistics. Hospitalization rates for primary diagnoses and job title/status with ≥2 documented cases were reported. Annual crude fatality rates per 100,000 full time equivalent workers, age ≥ 16 years, were estimated for 1990–2016 using annual average full time equivalent workers and the U.S. National Institute Occupational Safety and Health’s Employed Labor Force Query System as denominator.

**Results:**

Annual crude hospitalization rates decreased over time. Hospitalization and fatality rates were higher among young adult workers ages 19–21 years; non-Hispanic Whites; and, males. Percent fatality for ages 19–21 years was greater than ages 14–17 years and 18 years. Declines in hospitalization rates corresponded to decreases in reported injuries among career-technical-vocational education students. Age distribution varied slightly between hospital discharge data and New Jersey Safe Schools Program data.

**Conclusion:**

Hospitalization and fatality rates were higher among males than among females, possibly reflecting a tendency for males to engage in riskier jobs than females. Understanding injury disparities can inform public health prevention efforts. Trainings/interventions should aim at addressing the most frequently diagnosed conditions or nature of reported injuries, within those most impacted career clusters like sales/restaurant workers.

**Electronic supplementary material:**

The online version of this article (10.1186/s40621-019-0216-9) contains supplementary material, which is available to authorized users.

## Background

Injuries at work are a public health concern particularly among working adolescents (teens and youths, ages 14–21). Injuries contribute to temporary and permanent disabilities, reduced quality of life, increased mortality rates, and increased healthcare expenditure (Florence et al., 2015). Florence et al. estimated the total costs of injuries and violence in the United States in 2013 was about $671 billion; of this, costs associated with fatal injuries were $214 billion, and nonfatal injuries were $457 billion (Florence et al., 2015). According to the U.S. Centers for Disease Control and Prevention (CDC) (Centers for Disease Control and Prevention, 2017a), nearly 200,000 people die from injury each year—about one person every three minutes. In 2015, unintentional injury was the leading cause of death among persons aged one to 44 (Centers for Disease Control and Prevention, 2015a). Many injury survivors deal with life-long cognitive, physical, and financial outcomes directly or indirectly related to their injuries. In 2014, about 2.5 million people were hospitalized for injuries, and almost 27 million people were treated for injuries in an Emergency Department (ED) (Centers for Disease Control and Prevention, 2017b). Demographic disparities exist in the distribution of injury incidence and severity in diverse populations by age, gender, race, occupation/job title or industry, socioeconomic status indicators, and geographic factors (Centers for Disease Control and Prevention, 2015b; Laberge & Ledoux, 2011; Laberge et al., 2011; Centers for Disease Control and Prevention, 2003; Centers for Disease Control and Prevention, 2010; Shendell et al., 2010; Shendell et al., 2012a; Shendell et al., 2012b; Rubenstein et al., 2014; Apostolico & Shendell, 2016; Shendell et al., 2018).

About 80% of adolescents have worked at least one job prior to high school graduation (Centers for Disease Control and Prevention, 2015b). Each year, about 200,000 of these adolescents experience job related injury or illness, due in part to work place inexperience (unfamiliarity with the required tasks), lack of job-specific training, and/or lack of physical and/or emotional maturity (Centers for Disease Control and Prevention, 2015b; Laberge & Ledoux, 2011; Laberge et al., 2011; Centers for Disease Control and Prevention, 2003). Lack of knowledge about legal restrictions imposed on workers under age 18, including prohibited tasks and prohibited equipment under child labor laws, may also contribute to these adverse health outcomes. The National Institute for Occupational Safety and Health’s “Youth at Work: Talking Safety” curriculum (Centers for Disease Control and Prevention, 2010), notes how many positive traits of youth—energy, enthusiasm, and a need for increased challenge and responsibility—can cause adolescents to take on tasks they are not prepared to do safely, and/or make them reluctant to ask questions of their employers, managers, and/or supervisors.

Career-technical-vocational education (CTE) related injuries are mandated by law, under jurisdiction of New Jersey Department of Education (NJDOE), to be reported to the New Jersey Safe Schools Program (NJ SS) online reporting system. This is the only U.S. State law-based surveillance data for adolescents, a susceptible, vulnerable adolescent sub-population (Shendell et al., 2010; Shendell et al., 2012a; Shendell et al., 2012b; Rubenstein et al., 2014; Apostolico & Shendell, 2016; Shendell et al., 2018). To date, reported injuries are generally less severe, e.g., cases of permanent disability or death are rare (Shendell et al., 2010; Shendell et al., 2012a; Shendell et al., 2012b).

The NJ Department of Health’s (NJDOH) hospitalization and injury fatality records provide additional information about other CTE and non-CTE related injuries not reported to NJ SS but were captured due to required inpatient care. NJDOH data also include more severe and fatal injury cases, and other clinical diagnoses, across a wide spectra of career clusters, often requiring hospitalization periods.

This report examined NJDOH hospitalization and fatality records, in comparison with NJ SS surveillance reports, among injured adolescent workers. These analyses can inform future trainings of adolescent workers. These findings may also be applicable to established training programs including the “OSHA 10 Plus” in-person course, the NJDOE/NJ SS online 2-h portion introducing concepts of career, technical and vocational education safety and health and agency jurisdiction, and the “Career Cluster Specific PPE” online course of NJ SS, which are conducted in collaboration with the Rutgers School of Public Health Center for Public Health Workforce Development (Koshy et al., 2018).

## Methods

Details of the NJ SS online injury surveillance system, which covers secondary school students ages 14–21 in NJ approved CTE programs, are available online (New Jersey Safe Schools Program. Incident Reporting, 2019) as well as references cited (Shendell et al., 2010; Shendell et al., 2012a; Shendell et al., 2012b; Rubenstein et al., 2014; Apostolico & Shendell, 2016; Shendell et al., 2018).

Hospital discharge data (HDD) were used to obtain the annual number of work-related hospitalizations 2007–2016 for ages 14–24 years. HDD were obtained from the NJ Uniform Billing (UB) data (New Jersey Department of Health, 2018). HDD were abstracted based on age, state of residency, calendar year, and Workers’ Compensation as primary payer. To calculate the annual crude rate of hospitalizations per 100,000 employed persons, denominator data were obtained from the Bureau of Labor Statistics’ (BLS) Geographic Profile of Employment and Unemployment (U.S. Bureau of Labor Statistics (BLS), 2018). Estimated hospitalization rates were stratified by demographic data available, including age, gender, and race/ethnicity. Hospitalization rates for primary diagnoses and job title/status with two or more documented cases were also reported. Only primary diagnoses with at least two cases were included in detail in this report.

Fatalities among 14–24 year olds were obtained from the NJDOH Occupational Health Surveillance Unit’s Fatal Occupational Injuries Surveillance (FOIS) project. NJ FOIS is a project tracking fatal work-related injuries reported to have occurred in New Jersey. NJ FOIS data are used to examine factors contributing to those work-related fatal injuries, and to inform, develop and disseminate recommendations for preventing similar incidents in the future.

Annual crude fatality rates per 100,000 full time equivalent (FTE) workers, age 16 years and older, were estimated for 1990 to 2016 using annual average FTE workers age 16 years and older (U.S. Census Bureau, 2018) and NIOSH’s Employed Labor Force (ELF) Query System (Centers for Disease Control and Prevention, 2018), as the denominator. Fatality rates were also estimated by various demographic data available, industry and occupation (job title, status and industry classifications). The BLS data query tool (U.S. Bureau of Labor Statistics, 2018) was used to help stratify data by age group. Analyses were conducted using SAS version 9.4 (SAS [computer program], 2013).

This secondary analysis study was approved by Rutgers Biomedical and Health Sciences and Rutgers University-New Brunswick Institutional Review Board as part of the overall approval for the state-agency grant-funded NJ Safe Schools Program. This study was partially supported by the NJ Department of Education-Office of Career Readiness (for Rutgers School of Public Health authors).

## Results

There were 651 adolescent work-related injury cases identified using the NJ hospital discharge data (HDD) from 2007 to 2016. The NJ Safe Schools (NJ SS) Program online injury surveillance system identified 772 reported youth school-sponsored work-related injuries during the same time period (Additional file 1: Figure S1). Detailed descriptive and other statistics from the NJ SS online injury surveillance system (New Jersey Safe Schools Program. Incident Reporting, 2019) for the present study’s time period appear elsewhere in references cited (Shendell et al., 2010; Shendell et al., 2012a; Shendell et al., 2012b; Rubenstein et al., 2014; Apostolico & Shendell, 2016; Shendell et al., 2018). One hundred fourteen youth fatalities were identified from 1990 to 2016 through the NJ Fatal Occupational Injuries Surveillance project.

**Fig. 1:**
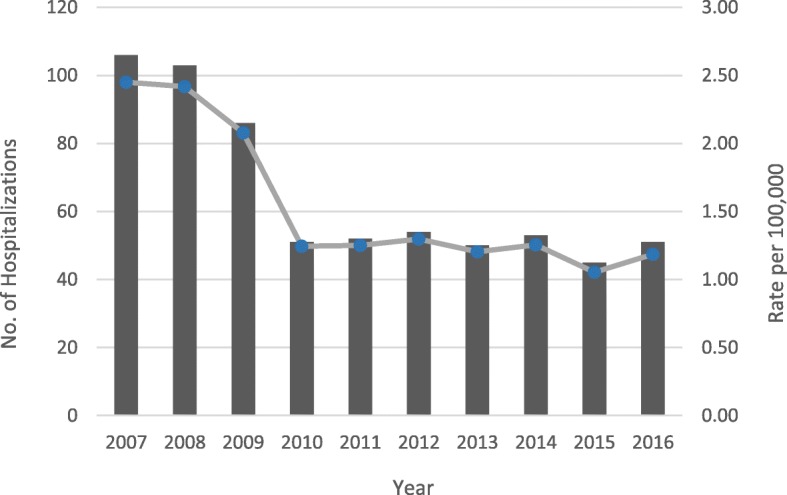
Annual Number and Crude Rate of Work-Related Youth Inpatient Hospitalizations in New Jersey by Year, 2007–2016

Annual crude inpatient hospitalization rates per 100,000 employed persons for youth workers gradually decreased between 2007 (2.45; 95% confidence interval (CI): 2.02, 2.95) and 2016 (Rate: 1.19; 95% CI: 1.06, 1.32). The largest decline in hospitalization rate was observed in 2010, a 40.4% decrease from 2009 (2.08, 95% CI: 1.67, 2.55 and rate: 1.24, 95% CI: 0.94, 1.62, respectively). However, rates were then similar from 2010 to 2016. (Fig. 1).

**Fig. 2:**
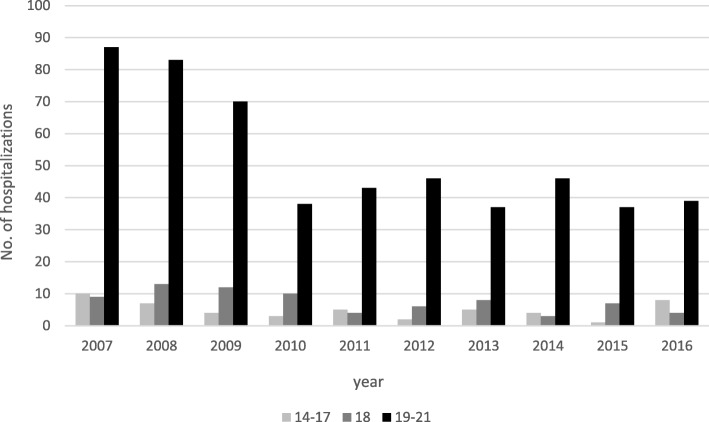
Number of Work-Related Youth Hospitalizations in New Jersey by Age, 2007–2016

Hospitalization counts between 2007 and 2016 were higher among those ages 19 to 21; workers 18 years of age recorded higher hospitalization counts than workers 14 to 17 years of age. Of the 651 hospitalizations recorded between 2007 and 2016, 526 (80.8%) were 19 to 21 years of age, 76 (11.7%) were 18 years old, while 49 (7.5) were 14 to 17 years of age. (Fig. 2).

Non-Hispanic youth workers had the largest hospitalization burden during the studied period of years (375, 57.6%); 230 (35.3%) Hispanic youth workers; and 46 (7%) workers of unspecified ethnicities were hospitalized. Non-Hispanic Whites had the largest hospitalization burden (422, 64.8%), followed by other races (147, 22.6%) and then Black or African Americans (47, 7.2%).

**Fig. 3:**
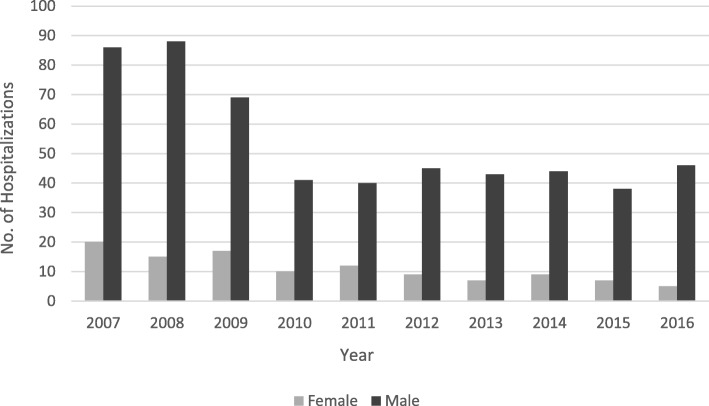
Work-Related Youth Hospitalizations in New Jersey by Gender, 2007–2016

During the study period, the hospitalization rate for males was five times higher than females (540, 83% and 111, 17%, respectively). While hospitalization rates for males remained higher than females during the study period, a noticeable decrease in male hospitalization was observed; female hospitalization counts also declined, since 2009. (Fig. 3).

The present analyses included injuries and illnesses, ranging from severe to fatal, from the 651 total inpatient hospitalizations. Type and body parts/location of injuries included: fractures (56, 8.6%), amputation (13, 2%), cellulitis and abscess of arm/foot (30, 4.6%), open wound in arm, knee or leg (14, 2%), concussion with loss of consciousness (14, 2%), and displacement of thoracic or lumbar intervertebral disc without myelopathy (12, 1.8%). Other categories, not largely represented, i.e., with less than 1% of reported injuries, included sprain of neck, knee or ankle, dehydration, and full-thickness skin loss.

**Fig. 4:**
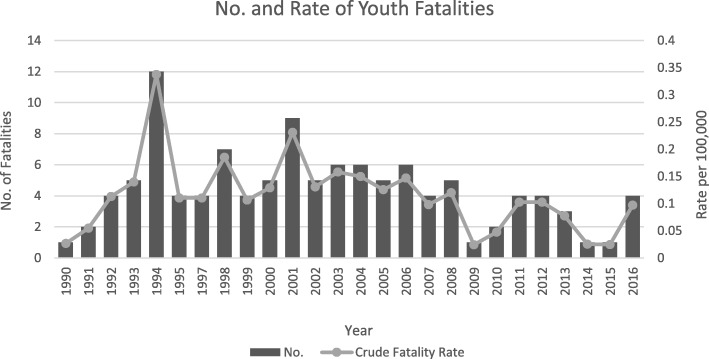
Annual Number and Crude Fatality Rate of Youth Workers in New Jersey, 1990–2016

Annual crude fatality rate per 100,000 FTE workers age 16 years and older has fluctuated, with 1994 and 2001 having higher rates than average (rate: 0.34, 95% CI: 0.18, 0.57 and rate: 0.23, 95% CI: 0.11, 0.42, respectively). (Fig. 4).

Hospitalization and fatalities were higher among adolescent workers ages 19 to 21 (Table 1). The percent fatality for the 19–21 age group is over three times the combined % fatality of the other two age groups, 14–17 and 18 (79.0, and 8.8% and 12.3% (or about 21%), respectively).

**Table 1:** Youth fatalities by age, 1990–2016

Age group	No.	%
< 18	10	8.77
18	14	12.28
19–21	90	78.95
Total	114	100

There were more fatal injuries among non-Hispanic youth workers than those of Hispanic origin, 64.9% (74) versus 32.5% (37); three (2.6%) are of unknown ethnicity. There was a wide racial disparity among hospitalized cases; non-Hispanic Whites recorded the most fatal cases, followed by blacks or African Americans (74.6 and 10.5%, respectively).

Job categories reported among hospitalized persons included construction (nine), sales/restaurant, automobile, landscape (five), technicians, laborers (25), members of school staff, and unemployed (41), among others. With respect to occupation (job title/industry classification), landscapers, construction laborers and restaurant workers recorded the most fatal cases (8.9, 7.0 and 6.1%, respectively).

## Discussion

Annual crude inpatient hospitalization rates gradually decreased over time; hospitalization and fatality rates were higher among adolescent workers between ages 19 and 21; non-Hispanic Whites had the greatest hospitalization and fatality rates; and, male inpatient hospitalization and fatality rates were higher than females.

Overall, the decline in hospitalization rates (Fig. 1) corresponds to a decrease in reported injuries among CTE students in NJ, as observed in NJ SS annual report. (Please see Additional file 1: Figure S1). The decrease in reported injuries in NJ SS online surveillance over time may be due to a combination of changes in vocational, CTE programs and courses, a switch from paper to online reporting, changes in the type of work students are placed in, underreporting, or actual declines in accident occurrence due to enhanced inspection and implementation of safety standards. Similar reasons could be directly applicable to NJDOH data.

Reporting of occupational injury-related hospitalization has been reported to be strongly influenced by the use of workers’ compensation benefit claims as primary payer (Shannon & Lowe, 2002; Rosenman et al., 2000). This workers’ compensation claim data underestimate the true burden of non-fatal work-related injuries. According to BLS and U.S. Department of Labor, the incidence of nonfatal occupational injuries in the U.S. declined steadily among U.S. private sector workers between 1990 and 2014 (Bureau of Labor Statistics, 2014; U.S. Department of Labor, 1999). This decline accompanied changes in occupational health and safety practices, such as increased identification of hazards and the institution of controls to mitigate dangers in the workplace. However, the observed decline in hospitalization rates could also be a result of limited access to healthcare or other underlying socioeconomic factors.

Calculated rates inherently vary based on differences in the denominator. NJ SS data are based on public secondary school CTE enrollment programs, while NJDOH data involves all adolescent workers in NJ, regardless of student/CTE enrollment status. The effect of this difference is an artificially smaller injury rate for NJ SS data compared to NJDOH data.

The age distribution was slightly different between hospitalization rates and injuries reported to NJ SS (Shendell et al., 2010; Shendell et al., 2012a). While persons ages 19 to 21 had higher hospitalization rates, persons ≤17 years of age had the most injury reports in NJ SS data. These data do not come as a surprise given the inexperience and insufficient job-specific trainings among adolescent workers. Data based on a mid-2016 update for the State of NJ by BLS provides some perspective of the demographics considered in this study (Bureau of Labor Statistics, 2014). About 22% of the state’s population are persons under age 18, i.e., about 1-in-5 New Jersey residents is school-aged, highlighting the importance of protecting this sub-population from work-related injuries. A high injury incidence might not necessarily translate into a high hospitalization rate, as is the case with the two age groups in the two separate reports discussed above. This is due to the differences in the severity scale between the NJ SS injury reports and the state hospitalization and fatality data. For example, it is possible some injuries reported to the NJ SS were severe enough to require a doctor’s or an emergency room’s visit but not severe enough to require hospitalization.

Racial disparity in injury distributions among injured youth workers has been previously reported, and non-Hispanic whites often bear much of the injury burden. This is similar to the finding that occupational injury rates were highest for non-black workers and lowest for black workers (Richardson et al., 2004). However, some studies have reported otherwise. For example, analysis of data from 1988 to 2000 from a cross-sectional longitudinal survey of youth workers in the U.S. indicated black and Hispanic workers (both men and women) experienced a higher injury burden than non-Hispanic white men (Strong & Zimmerman, 2005). There may be unique demographics among NJ adolescents. For example, non-Hispanic whites may constitute the largest proportion of the NJ youth subpopulation among working adolescents, and this may be related to the observed larger number of hospitalizations among non-Hispanic whites. The differences observed by race-ethnicity could also reflect differences in types of jobs worked, in terms of their hazardous nature, and the degree of injury risk. For instance, Strong and Zimmerman (Strong & Zimmerman, 2005) reported Blacks were more likely employed as machine operators, fabricators, and laborers, while non-Hispanic white men typically worked less hazardous jobs including managerial positions. A 2006 cross-sectional study observed how Hispanic and black teens (compared with non-Hispanic white teens) had an adjusted odds ratio of more missed days of work due to injury/illness of 3.1 and 2.3, respectively (Zierold & Anderson, 2006). These authors also reported a higher adjusted odds ratio of general injury and severe injury among blacks (Zierold & Anderson, 2006). According to the U.S. Census data for NJ, about 70% of the population is white, about 15% of residents are blacks or African Americans, and about 1-in-10 (9.8%) residents is Asian (United States Census Bureau. QuickFacts: New Jersey, 2017). This explains in part how racial demographics in the State of NJ might have impacted injury distribution among youth workers.

The observed high male hospitalization and fatality rates might be suggestive of higher tendency for males to engage in more risky jobs than females or a reflection of the currently reported gender distribution in the underlying population. Similarly, annual reports from the NJ SS injury surveillance data suggested injury rates are disproportionately higher among males than among females (Shendell et al., 2010; Shendell et al., 2012a; Shendell et al., 2012b).

The distribution of injuries in this report were similar in some respects to the NJ SS injury reports, where cuts/lacerations (historically about 2-in-5 of reported injuries but up to 52.4% in 2016–2017) and “caught in, under or between” (historically about 1-in-5 of reported injuries including in 2016–2017) were the most frequently reported injury among career and technical education students, with only one reported amputation in several years (Shendell et al., 2012b). This was supported by an overview of NJDOH syndromic surveillance data involving ED visits. Prevalence estimates of the nature of injury are not available for direct comparison in this report. However, the prevalence of more severe injuries was lower in the NJ SS report than the HDD and fatality data. The recent NJ SS injury report to NJDOE stated about 53% of reported cases visited hospital emergency rooms for treatment, while 47% made doctor visits (Uhiara & Shendell, 2017). In NJ SS injury reports, many reported cases involved students working in “hazardous” career clusters, the majority of which were architecture and construction (60%). Among “non-hazardous” career clusters, many reported cases fell under hospitality and tourism (55.6%) (Shendell et al., 2016). Trainings/interventions should aim at addressing most frequently diagnosed conditions or nature of injury, or career clusters such as sales/restaurant workers. While some of those clusters may be beyond the current NJ SS scope of training, a collaboration with NJDOH and delivery of such trainings would be of public health relevance.

This study had known limitations. One limitation of this report is the lag time in availability of the denominator data as well as both BLS and UB data. Thus, estimated rates were only calculated through 2016. Also, the statistics provided in this study are crude rates, which may not show the true burden of deaths in a particular group and may not take age or sex into account. Another limitation is HDD may be more likely to include more severe injuries requiring hospitalization and may miss less severe injuries of public health importance, including workplace violence-related injuries. Furthermore, occupational injuries and illnesses are often underreported since individuals sometimes do not file workers’ compensation claims and some individuals such as those who are self-employed like farmers and independent contractors are not covered by workers’ compensation.

## Conclusions

Injuries and its related problems create a substantial economic and health burden in the United States, especially injury among youth. Understanding the causes and mechanisms of injuries creating the largest share of this burden, and understanding disparities among affected groups, can inform and improve public health prevention efforts. Injury occurrence is largely determined by environmental as well as behavioral characteristics. Modification of environmental characteristics in an effort to combat work place injuries always seems more feasible than individual behavioral alterations. As a result, many proposed solutions and practical attempts to scale down injuries targeted environmental modifications and have ignored behavioral modification approaches. Failure to recognize the difficulty of improving individual behavior has often led to failure to apply more effective alternative counter measures to injury. Thus, to more effectively tackle injury as a public health problem, a holistic approach must be adopted, which targets the physical, behavioral and environmental/occupational components. This would require an effective collaboration among stakeholders involved in injury surveillance, treatment, education, trainings, etc. For example, the collaboration among NJ SS, NJ DOH and other certified health and safety trainers/educators, behavioral experts, and school administrators. New trainings or improvements to existing trainings could be effective at reducing the prevalence of injury occurrence among youth workers, such as enhancing the in-person and online courses of NJ SS at Rutgers School of Public Health and agency partners (Koshy et al., 2018; Shendell et al., 2017). This is an example of using injury surveillance data as lagging indicators in as near real-time as possible, versus using leading indicators, to address and reduce risks in workplaces (Straub, 2018a; Straub, 2018b).

Health and safety education is an important component of injury prevention for employed youth. In addition to workplace-specific training, young people also need the opportunity to learn and practice general health and safety skills applicable to other jobs in the event of job changes. Youth workers should be adequately trained on hazard identification, control and elimination; what to do in emergency situations; what rights they have on the job; and, how to speak up and raise safety concerns effectively when problems arise at work.

## Additional file

Additional file 1: **Figure S1.** Number of Injury Incidents Reported By Fiscal Year within the New Jersey Safe Schools Program Surveillance System (Shendell et al., 2010; Shendell et al., 2012a; Shendell et al., 2012b; Rubenstein et al., 2014; Apostolico & Shendell, 2016; Shendell et al., 2018; New Jersey Safe Schools Program. Incident Reporting, 2019) (DOC 178 kb)

## Data Availability

This study’s data are secured on computers per IRB approved stewardship of NJ SS and/or are also publicly available from NJDOE. Datasets used and analyzed during the current study are available from the corresponding author on reasonable request.

## Abbreviations

BLS: U.S. Bureau of Labor Statistics
CDC: U.S. Centers for Disease Control and Prevention
CI: confidence interval
CTE: Career-technical-vocational education
FTE: Full-time equivalent
HDD: Hospital Discharge Data
HR: Hospitalization Rate
NJ SS: New Jersey Safe Schools Program
NJ: New Jersey
NJDOE: New Jersey Department of Education
NJDOH: New Jersey Department of Health
UB: Uniform billing
