# Supplementary material for: Reported injury, hospitalization, and injury fatality rates among New Jersey adolescent workers
Source: Inj Epidemiol. 2019 Aug 19;6:37. doi: 10.1186/s40621-019-0216-9 (PMC6699110; doi:10.1186/s40621-019-0216-9)

**Additional file 1: Figure S1** Number of Injury Incidents Reported By Fiscal Year within the New Jersey Safe Schools Program Surveillance System^10-15, 17^


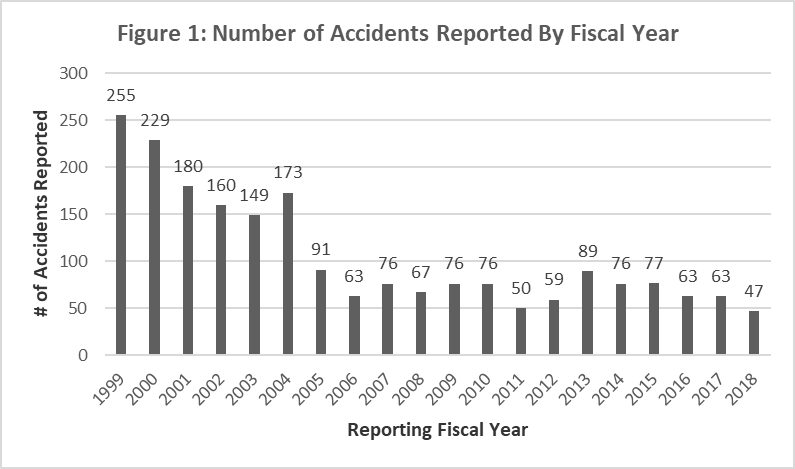

Supplement: Supplementary file 1 — Figure S1. Number of Injury Incidents Reported By Fiscal Year within the New Jersey Safe Schools Program Surveillance System (Shendell et al., 2010; Shendell et al., 2012a; Shendell et al., 2012b; Rubenstein et al., 2014; Apostolico & Shendell, 2016; Shendell et al., 2018; New Jersey Safe Schools Program. Incident Reporting, 2019) (DOC 178 kb) [file 40621_2019_216_MOESM1_ESM.doc]
